# Genetic susceptibility to juvenile idiopathic arthritis in Iranian children

**DOI:** 10.1186/1546-0096-9-S1-P103

**Published:** 2011-09-14

**Authors:** Reza Shiari, Shirin Farivar, Elham Hadia

**Affiliations:** 1 Shahid Beheshti University of Medical Sciences, Mofid Children Hospital, Tehran-Iran; 2 Pediatric Infectious Research Center (PIRC), Tehran-Iran; 3 Faculty of Biosciences, Shahid Beheshti University, GC. Tehran-Iran

## Background

Juvenile idiopathic arthritis (JIA) is the most common chronic rheumatic disease in children. It is a clinical diagnosis in a child, less than16 years of age with arthritis for at least 6 weeks duration and exclusion of other causes of arthritis.

## Aims

This study was comprised of Iranian children with oligoarthritis and rheumatoid factor negative (RF) polyarthritis subtypes of juvenile idiopathic arthritis (JIA) to determine the association of HLA-DRB1 alleles in Iranian population.

## Methods

HLA-DRB1 alleles were investigated in 33 Iranian children with oligoarthritis and RF negative polyarthritis JIA and compared with 45 healthy controls. HLA typing was performed by PCR with sequence specific primers in either of the two groups and followed by direct detection of HLA polymorphism by sequence analysis in patient group.

## Results

HLA-DRB1*11 was found to be the most frequent allele associated with oligoarthritis and RF negative polyarthritis JIA in Iran followed by DRB1 *08. The frequencies of HLA-DRB1*04 and *13 were not significantly different in both groups.

## Conclusions

We concluded that there was a significant difference in allele frequencies between patients and control group that may help in predicting the susceptibility to oligoarthritis and rheumatic factor negative polyarthritis JIA.

